# Regulation of *FpvelC* on Conidiation, Pathogenicity and Secondary Metabolism in *Fusarium proliferatum*

**DOI:** 10.3390/toxins17090433

**Published:** 2025-08-30

**Authors:** Ling Wang, Shaoqing Tang, Weiyang Liao, Zhonghua Sheng, Shikai Hu, Gui’ai Jiao, Gaoneng Shao, Lihong Xie, Peisong Hu

**Affiliations:** State Key Laboratory of Rice Biology and Breeding, China National Center for Rice Improvement, China National Rice Research Institute, Hangzhou 311401, China; tangshaoqing@caas.cn (S.T.); 82101215336@caas.cn (W.L.); shengzhonghua@caas.cn (Z.S.); hushikai@caas.cn (S.H.); jiaoguiai@caas.cn (G.J.); shaogaoneng@caas.cn (G.S.); xielihong@caas.cn (L.X.)

**Keywords:** *Fusarium proliferatum*, velvet protein, conidiation, pathogenicity, fumonisins

## Abstract

The velvet complex is a master regulator of multiple physiological processes in filamentous fungi. In this study, we characterized the functions of velvet gene *FpvelC* in *Fusarium proliferatum*, which was the causative agent of rice spikelet rot disease. Compared with the wild-type Fp9 strain, deletion of *FpvelC* hindered conidiation, leading to a low level of trehalose content but excessive accumulation of chitin in conidia. Lack of *FpvelC* resulted in increased sensitivity to oxidative stress and decreased expression of antioxidant genes. Notably, Δ*FpvelC* exhibited attenuated pathogenicity on rice and maize, failure to produce invasive hyphae, and downregulation of genes encoding xylanases and xyloglucanases during infection processes. Nevertheless, disruption of *FpvelC* enhanced production of fumonisin B1 (FB1) and fusaric acid concomitantly; transcripts of the clustering genes responsible for the two mycotoxins’ biosynthesis were significantly increased. Additionally, the absence of *FpvelC* was displayed as more sensitive to rapamycin than the Fp9 strain, accompanied with less intracellular glutamine. Overall, *FpvelC* played versatile roles in conidiation, response to oxidative stress, pathogenicity and mycotoxins production in *F. proliferatum*.

## 1. Introduction

*Fusarium proliferatum* (Matsushima) Nirenberg is a worldwide fungal pathogen, which can infect a wide range of important crops, including rice, wheat, maize, barley, oat, rye, millet and sorghum [1]. As a polyphagous fungus, *F. proliferatum* is able to survive on various crops for long periods, and it has the extraordinary adaptation ability of colonizing new environments. Under favorable climatic conditions, the infection often leads to substantial economic losses. More importantly, colonization of hosts by *F. proliferatum* impairs the security and quality of agricultural products due to mycotoxins contamination. The pathogen is a main producer of polyketide-derived fumonisins [2]. To date, more than 28 structural analogs of fumonisins have been identified, with fumonisin B1 (FB1) occupying a large proportion, with a percentage of over 70% [3]. FB1 can cause leukoencephalomalacia in horses, pulmonary edema syndrome in pigs and hepatic and renal damage in rodents [4]. High levels of FB1 exposure are associated with outbreaks of esophagus cancer and neural tube defects in humans, as evidenced from epidemiological surveys [5]. Strict legislations have been set to control the maximum acceptable levels for FB1 in food and feedstuffs by the European Commission, the World Health Organization, the Food and Agriculture Organization and the Codex Alimentarius Commission [6]. In addition to FB1, *F. proliferatum* can produce some other mycotoxins, such as fusarin acid, fusarin C, fusarubin, beauvericin, moniliformin and fusaproliferin [7]. Among them, fusaric acid, a pyridine carboxylic acid derivative, not only has strong phytotoxic properties to plants [8], but also exerts mild toxicity towards animals, causing neurotoxicity in rats and notochord malformation in zebrafish [9,10]. Cytotoxicity of fusaric acid is closely linked to mitochondrial dysfunction, DNA hypomethylation, cell apoptosis, membrane permeability and oxidative damage [11,12]. With respect to the economic and public health issues, it is necessary to take effective approaches to minimize or remove mycotoxins contamination in agricultural and food products.

In filamentous fungi, velvet family proteins consist of a class of regulatory proteins harboring a common velvet domain [13]. Four well-known members, veA (velvet A), velB (velvet-like B), velC (velvet-like C) and vosA (viability of spores A), have been characterized in the model organism *Aspergillus nidulans* [13,14]. Velvet proteins and laeA usually form a variety of homodimers, heterodimers or heterotrimers, such as velB-vosA, velC-vosA, velB-velB and velB-veA-laeA, thus participating in the regulation of secondary metabolites [15]. In toxin-producing fungi, veA was reported to be indispensable for the synthesis of sterigmatocystin in *Aspergillus pachycristatus* [16], aflatoxin B1 in *Aspergillus flavus* [17], ochratoxin A in *Aspergillus ochraceus* [18], alternariol in *Alternaria alternata* [19], and trichothecene in *Fusarium graminearum* [20]. VelB served as a positive activator for deoxynivalenol in *F. graminearum* [21] and *Fusarium pseudograminearum* [22] or for patulin in *Penicillium expansum* [23], whereas it negatively regulated the production of pigment melanin in *Colletotrichum siamense* [24]. However, the roles of velC and vosA proteins remain poorly defined in toxigenic fungal species.

Rice (*Oryza sativa* L.) is one of the most widely cultivated crops in the world. Rice spikelet rot disease (RSRD) caused by *F. proliferatum* is one of the prevalent fungal diseases in China [25]. Severe epidemics have occurred frequently under humid conditions in the main rice-growing regions, with estimated yield losses of up to 30% [25]. Meanwhile, the mycotoxins produced by the pathogen are harmful to the health of humans and animals. Chemical fungicides have been routinely used to manage RSRD due to lack of resistant rice cultivars [26]. Understanding the processes of biology and metabolism of *F. proliferatum* is crucial, as it is instrumental for the prevention and treatment of RSRD. The purpose of this study was to investigate the biological functions of *velC* orthologous (*FpvelC*) in *F. proliferatum*. Our findings indicate that *FpvelC* played key roles in asexual development, virulence and secondary metabolism. This was the first functional study to reveal the role of *FpvelC* in pathogenesis and mycotoxins biosynthesis in *F. proliferatum*.

## 2. Results

### 2.1. Identification and Deletion of FpvelC

**Figure 1:**
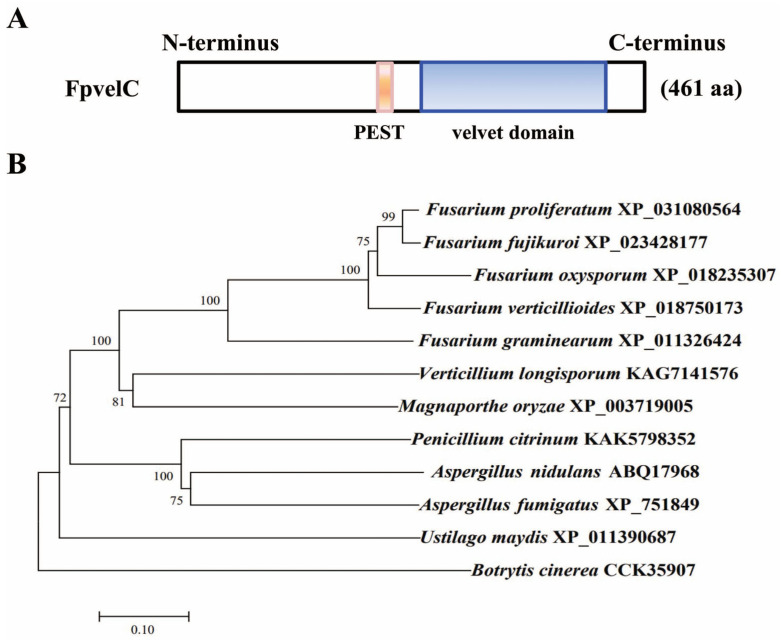
The structure and phylogenetic tree of *F. proliferatum* FpvelC protein. (**A**) Domain architecture of the FpvelC protein in *F. proliferatum*. The length of amino acid was noted in parentheses. Blue indicates a velvet domain between amino acids 245 and 426, while pink indicates a PEST domain between amino acids 198 and213. (**B**) Phylogenetic analysis of FpvelC protein and the orthologs in different filamentous fungi. A phylogenetic tree was constructed based on the amino acid sequences using MEGA 11 using the neighbor-joining method with 1000 bootstrap repetitions. Numbers on the branches represent the percentage of replicates supporting each branch. The fungal species and GenBank accession numbers are labeled on the right.

The FpvelC protein was obtained from the genome of *F. proliferatum* through a BLASTP search using the velC ortholog of *A. nidulans* (ABQ17968) as a bait. Analysis of conserved domains showed that FpvelC contained a velvet domain at the C-terminus. The EMBOSS program identified a potential proline, glutamate, serine and threonine (PEST) region, which was a signal peptide for protein degradation (Figure 1A). Phylogenetic analysis indicated that the velC protein was evolutionarily conserved across filamentous ascomycota, and the FpvelC protein exhibited the closest similarity to homologous proteins of *Fusarium* species (Figure 1B).

To further clarify the roles of the *FpvelC* gene in *F. proliferatum*, a deletion mutant and a complementary strain were constructed by homologous recombination. Deletion mutant Δ*FpvelC* was generated by replacing the open reading frame (ORF) with a hygromycin resistance cassette (*HYG*) (Figure S1A). Positive transformants were verified by diagnostic PCR and Southern blot analysis (Figure S1B,C). Upstream and downstream regions of homologous recombination were further confirmed by Sanger sequencing. The full-length sequence of the *FpvelC* gene including its promoter region was reintroduced into Δ*FpvelC* to generate a complementary strain Δ*FpvelC*-C (Figure S2).

### 2.2. FpvelC Is Indispensable for Normal Conidiation

**Figure 2:**
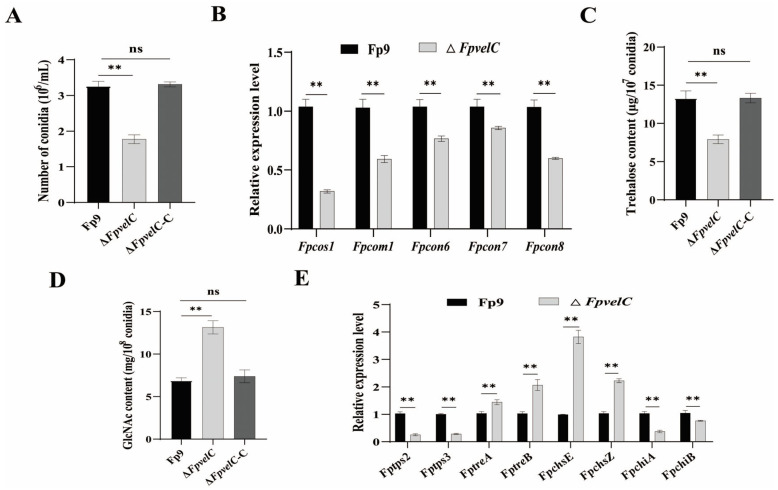
Influence of *FpvelC* on conidiation in *F. proliferatum*. (**A**) The number of conidia of Δ*FpvelC* in YEPD liquid media. (**B**) Relative expression levels of conidiation-related genes in conidia of Δ*FpvelC* after being cultured in YEPD media for 48 h. *Fpcos1* and *Fpcom1* genes encoded transcription factors; *Fpcon6*, *Fpcon7* and *Fpcon8* genes encoded conidiation-specific proteins. (**C**) The amounts of trehalose per 10^7^ conidia of Δ*FpvelC*. (**D**) The amounts of *N*-acetylglucosamine (GlcNAc) per 10^8^ conidia of Δ*FpvelC*. (**E**) Relative expression levels of genes associated with trehalose and chitin synthesis in conidia of Δ*FpvelC* after being cultured in YEPD media for 48 h. The *Fptps2* and *Fptps3* genes encoding alpha, alpha-trehalose phosphate synthases were involved in trehalose synthesis; the *FptreA* gene encoding alpha, alpha-trehalose glucohydrolase and the *FptreB* gene encoding trehalase were involved in trehalose hydrolysis; the *FpchsE* and *FpchsZ* genes encoding chitin synthases were involved in chitin synthesis; and the *FpchiA* gene encoding class III chitinase and the *FpchiB* gene encoding class V chitinase were involved in chitin degradation. The bars represent the standard deviation from three replicates. Significant differences were determined by Student’s *t*-test (**, *p* < 0.01; ns, not significant). All experiments were repeated three times with three replicates each time.

To elucidate the effect of *FpvelC* in asexual sporulation, all strains (Fp9, Δ*FpvelC* and Δ*FpvelC*-C) were cultured in yeast extract peptone dextrose (YEPD) liquid media. Compared with the Fp9 strain, the number of conidia was significantly lower in Δ*FpvelC* (Figure 2A). Transcript levels of sporogenesis-related genes, including *Fpcos1*, *Fpcom1*, *Fpcon6*, *Fpcon7* and *Fpcon8*, were decreased in Δ*FpvelC* (Figure 2B). In conidia, trehalose amount was less in Δ*FpvelC* than that of the Fp9 strain, but chitin content was more in Δ*FpvelC* than that of the Fp9 strain (Figure 2C,D). The mRNA levels of genes associated with trehalose synthesis (*Fptps2* and *Fptps3*) and chitin degradation (*FpchiA* and *FpchiB*) were downregulated, whereas the transcripts of genes involved in trehalose hydrolysis (*FptreA* and *FptreB*) and chitin synthesis (*FpchsE* and *FpchsZ*) were markedly elevated in the conidia of ∆*FpvelC* (Figure 2E). The reversal of conidiation phenomena was observed after reintroducing the *FpvelC* gene into the ∆*FpvelC* mutant. Collectively, these results demonstrate that *FpvelC* played a critical role in conidiation of *F. proliferatum*.

### 2.3. FpvelC Is Required for Responses to Stress Tolerance

**Figure 3:**
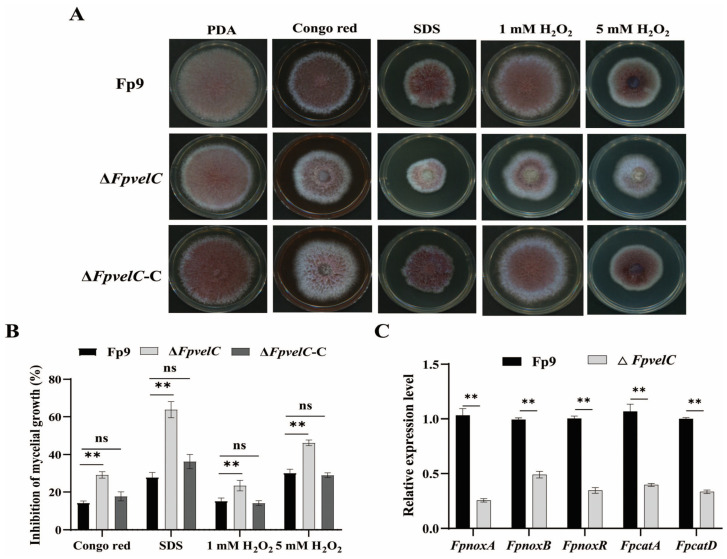
Effect of *FpvelC* on stress responses in *F. proliferatum*. (**A**) Colony phenotypes of Δ*FpvelC* on PDA media supplemented with 0.3 mg/mL Congo red, 0.01% SDS, or 1 mM or 5 mM H_2_O_2_ at 28 °C for 5 days. (**B**) The inhibition of mycelial growth of Δ*FpvelC* under different stresses. (**C**) Relative expression levels of antioxidant genes in Δ*FpvelC*. After being grown in PDB media for 3 days, Δ*FpvelC* was treated with 5 mM H_2_O_2_ for 1 h. *FpnoxA*, *FpnoxB* and *FpnoxR* encoded NADPH oxidases; *FpcatA* and *FpcatD* encoded catalases. The bars represent the standard deviations from three replicates. Significant differences were determined by Student’s *t*-test (**, *p* < 0.01; ns, not significant). All experiments were repeated three times with three replicates each time.

To evaluate the involvement of *FpvelC* in response to abiotic stimuli, all strains were cultured on potato dextrose agar (PDA) media supplemented with chemical reagents. Compared with the Fp9 strain, the growth of Δ*FpvelC* was significantly delayed when exposed to the cell wall-perturbing agents Congo red and cell membrane inhibitor sodium dodecyl sulphate (SDS) (Figure 3A,B). Furthermore, the relative inhibition of growth in Δ*FpvelC* was obviously higher than that of the Fp9 strain after being treated with different concentrations of hydrogen peroxide (H_2_O_2_), suggesting that Δ*FpvelC* was more sensitive to oxidative stress (Figure 3A,B). The levels of mRNA from oxidative responsive genes, NADPH oxidase subfamilies (*FpnoxA*, *FpnoxB* and *FpnoxC*) and catalase genes (*FpcatA*, *FpcatD*) were strongly repressed in Δ*FpvelC* after exposure to H_2_O_2_ (Figure 3C). Reintroduction of the *FpvelC* gene into Δ*FpvelC* rescued the stress tolerance. Taken together, these results imply that *FpvelC* was involved in maintenance of cell wall integrity and resistance to oxidative stress in *F. proliferatum*.

### 2.4. FpvelC Plays an Important Role in Full Virulence

**Figure 4:**
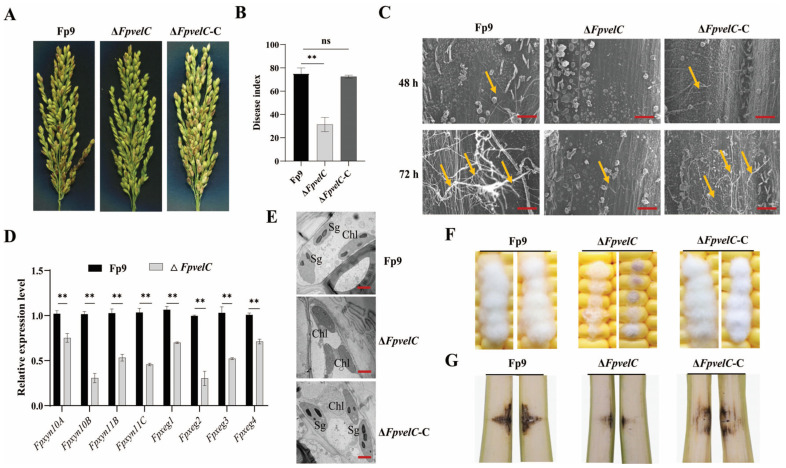
Involvement of *FpvelC* in the pathogenicity of *F. proliferatum*. (**A**) Disease symptoms of rice spikelets inoculated with Δ*FpvelC* at 21 dpi. (**B**) Disease index of rice spikelets inoculated with Δ*FpvelC* at 21 dpi. (**C**) Invasive hyphae of Δ*FpvelC* on the epidermis of rice glumes under scanning electron microscopy at 48 hpi and 72 hpi. Infectious hyphae are denoted with yellow arrows. Scale bars = 100 μm. (**D**) Relative expression levels of the genes encoding xylanases and xyloglucanases in infected glumes challenged by Δ*FpvelC* at 72 hpi. *Fpxyn10A*, *Fpxyn10B*, *Fpxyn11B* and *Fpxyn11C* genes encoded xylanases, and *Fpxeg1*, *Fpxeg2*, *Fpxeg3* and *Fpxeg4* genes encoded xyloglucanases. (**E**) Ultrastructure of rice glumes inoculated with Δ*FpvelC* under transmission electron microscopy at 72 hpi. Sg represents starch grain. Chl represents chloroplast. Scale bars = 2 μm. (**F**) Disease symptoms of maize kernels inoculated with Δ*FpvelC* at 7 dpi. (**G**) Disease symptoms of maize stalks inoculated with Δ*FpvelC* at 14 dpi. The bars represent the standard deviation from three replicates. Significant differences were determined by Student’s *t*-test (**, *p* < 0.01; ns, not significant). All experiments were repeated three times with three replicates each time.

To detect the potential function of *FpvelC* in plant colonization, susceptible rice and maize were inoculated with the conidial suspension of each strain. When infected with the Fp9 strain, rice spikelets exhibited wilting and rotten symptoms, whereas Δ*FpvelC* caused minor necrosis on spikelets at 21 days post-inoculation (dpi) (Figure 4A). The disease index of Δ*FpvelC* was much less than that of the Fp9 strain (Figure 4B). During the infection processes, the Fp9 strain developed copious hyphae along the epidermal cells of rice glumes at 48 h post-inoculation (hpi), and numerous hyphae gathered together at 72 hpi. Conversely, the fungal colonization was rarely detected in Δ*FpvelC*-inoculated glumes at 48 hpi, and sparse invasive hyphae were attached to the surfaces of glumes at 72 hpi (Figure 4C). Transcript levels of genes encoding xylanases and xyloglucanases of *F. proliferatum* were significantly decreased in Δ*FpvelC*-inoculated plants (Figure 4D). At the same time, starch grains of chloroplasts in glumes challenged with Fp9 appeared augmented, while starch grains in Δ*FpvelC*-infected glumes were obviously fewer (Figure 4E). In the case of corn infection assays, maize treated with the Fp9 strain showed dense aerial hyphae on kernels and necrotic lesions in stalks, but lesions were relatively smaller after being inoculated with Δ*FpvelC* (Figure 4F,G). The ability of Δ*FpvelC* to infect hosts was restored after complementation with a wild-type copy of *FpvelC*. These combined data substantiate that *FpvelC* contributed to pathogenicity in the initiation of infection events.

### 2.5. FpvelC Negatively Governs Production of Secondary Metabolites

**Figure 5:**
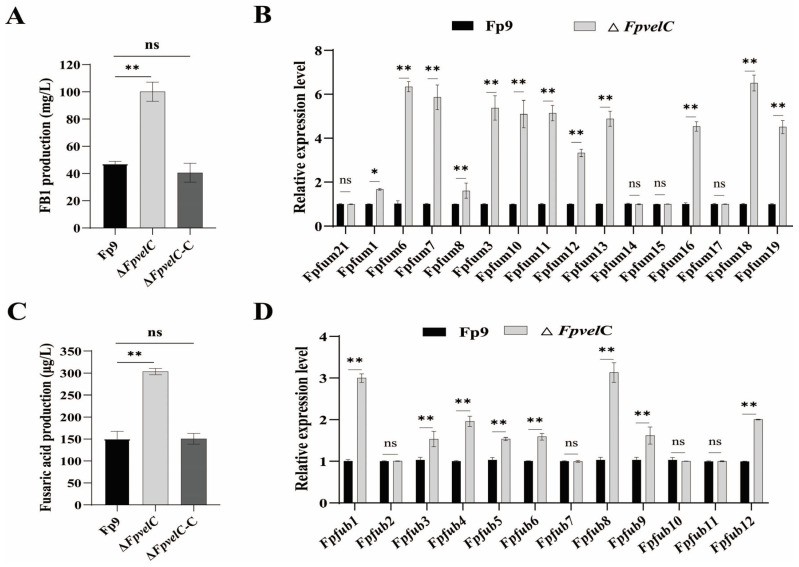
Impact of *FpvelC* on production of FB1 and fusaric acid in *F. proliferatum*. (**A**) Quantification of FB1 produced by Δ*FpvelC* in PDB cultures at 28 °C for 10 days. (**B**) Relative expression levels of genes (*Fpfums*) responsible for fumonisin biosynthesis in Δ*FpvelC* after being cultured in PDB for 72 h. (**C**) Quantification of fusaric acid produced by Δ*FpvelC* in PDB cultures at 28 °C for 10 days. (**D**) Relative expression levels of genes (*Fpfubs*) responsible for fusaric acid biosynthesis in Δ*FpvelC* after being cultured in PDB for 72 h. The bars represent the standard deviation from three replicates. Significant differences were determined by Student’s *t*-test (*, *p* < 0.05; **, *p* < 0.01; ns, not significant). The experiments were performed in triplicate with three replicates each time.

In order to determine if *FpvelC* participated in specialized metabolism, metabolite extracts from cultures of each strain in potato dextrose broth (PDB) were measured by high-performance liquid chromatography–tandem mass spectrometry (HPLC-MS/MS). Evidently, Δ*FpvelC* yielded greatly increased amounts of FB1 when compared with the Fp9 strain (Figure 5A). Simultaneously, levels of mRNA from most genes (*Fpfums*) involved in fumonisin biosynthesis were dramatically higher in Δ*FpvelC* than those in the Fp9 strain (Figure 5B). Meanwhile, production of fusaric acid was markedly elevated in Δ*FpvelC* cultures (Figure 5C). Correspondingly, expressions of the majority of backbone genes (*Fpfubs*) responsible for fusaric acid biosynthesis were upregulated in Δ*FpvelC* (Figure 5D). Reintroduction of the intact *FpvelC* allele into Δ*FpvelC* fully restored the levels, resembling those observed in the Fp9 strain. These results suggest that *FpvelC* played negative roles in regulating production of fumonisins and fusaric acid in *F. proliferatum*.

### 2.6. FpvelC Is Involved in Nitrogen Metabolism

**Figure 6:**
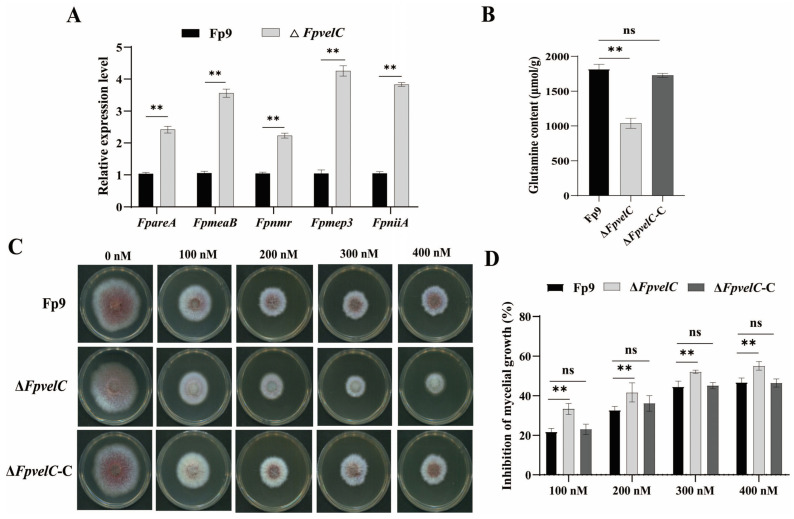
The role of *FpvelC* in nitrogen metabolism in *F. proliferatum*. (**A**) Relative expression levels of genes associated with nitrogen regulation in Δ*FpvelC*. The *FpareA* gene encoded the GATA transcription factor, the *FpmeaB* gene encoded the bZIP transcription factor, the *FpnmrA* gene encoded the NmrA family protein, the *Fpmep3* gene encoded ammonium permease, and the *Fpnii1* gene encoded nitrite reductase. (**B**) Quantification of intracellular glutamine in Δ*FpvelC*. (**C**) Colony morphology of Δ*FpvelC* on MM media amended with TOR kinase inhibitor rapamycin at final concentrations of 100, 200, 300 or 400 nM at 28 °C for 4 days. (**D**) The inhibition of mycelial growth of Δ*FpvelC* treated with different concentrations of rapamycin. The bars represent the standard deviation from three replicates. Significant differences were determined by Student’s *t*-test (**, *p* < 0.01; ns, not significant). The experiments were performed in triplicate with three replicates each time.

To ascertain whether disruption of *FpvelC* affected nitrogen metabolism, expression of genes involved in nitrogen regulation was investigated in Δ*FpvelC*. Relative to the Fp9 strain, mRNA levels of nitrogen regulators (*FpareA*, *FpmeaB* and *Fpnmr*), ammonium permease (*Fpmep3*) and nitrate reductase (*FpniiA*) were markedly increased in Δ*FpvelC* (Figure 6A). The amount of intracellular glutamine was dramatically decreased in Δ*FpvelC* (Figure 6B). Moreover, Δ*FpvelC* was more sensitive to a series of concentrations of rapamycin than the Fp9 strain, when grown in minimal medium (MM) media with glutamine as a sole nitrogen source (Figure 6C,D). These results illustrate that *FpvelC* might be implicated in the nitrogen-sensing pathway.

## 3. Discussion

*F. proliferatum* is notorious for its capability of producing mycotoxins, which threaten the health of humans and domestic animals [27]. A diverse array of corn and corn-based food commodities were frequently contaminated by *F. proliferatum* due to growth of toxigenic fungi and/or mycotoxins production, especially in tropical and subtropical regions of Europe, Asia and North Africa [28,29]. Considering economic losses and food safety, it is urgent to develop promising drug targets for control strategies to prevent *F. proliferatum* infection and mycotoxin contamination in agricultural products. Velvet complex has been known to be critical for fungal development and differentiation [15]. The present study firstly characterized the functions of velvet gene *FpvelC* in pathogenicity and secondary metabolism in *F. proliferatum*.

Asexual spores (conidia) are the main propagules of filamentous fungi. Infection of *F. proliferatum* on crops was sustained by dissemination and adaptation of conidia in ecological niches. Absence of *FpvelC* resulted in decreased conidiation in *F. proliferatum*. Notably, transcript abundances of sporulation-related genes were reduced in Δ*FpvelC*, including *Fpcos1*, *Fpcom1*, *Fpcon6*, *Fpcon7* and *Fpcon8* (orthologs of *cos1*, *com1*, *con6*, *con7* and *con8* in *Neurospora crassa*). Of these, *com1* and *con7* genes governed phialides differentiation, while *cos1*, *con6* and *con8* genes were responsible for the formation and maturation of conidia [30,31]. Especially, *con7* played a crucial role in controlling the expression of transcription factor *abaA* of the central developmental pathway for conidiation [32,33]. These results infer that *FpvelC* acted as a positive activator in conidiation during vegetative growth in *F. proliferatum*. This mode of action was similar to *velC* orthologs in other pathogenic fungi, including *Verticillium dahliae* [34], *Ustilaginoidea virens* [35] and *Magnaporthe oryzae* [36]. By contrast, there were several reports that *velC* negatively regulated conidiation in *A. nidulans* [37], *Penicillium chrysogenum* [38] and *Fusarium oxysporum* [39]. Apart from alteration in expression patterns of conidiation-specific genes, another reason for impaired conidiation might be attributed to the perturbed conidia structure. Deletion of *FpvelC* resulted in less trehalose through interference with trehalose synthesis in conidia, whereas accumulation of chitin was opposite to trehalose behaviors in Δ*FpvelC*. Moreover, Δ*FpvelC* displayed higher sensitivity towards cell wall stresses. The trehalose was a non-reducing disaccharide that was conducive to conidial viability and stress tolerance [40]. The chitin was the primary polysaccharide of spore walls, which was indispensable for cell wall integrity and rigidity [40]. Therefore, loss of *FpvelC* led to damage to the cell wall by influencing the synthesis and distribution of trehalose and chitin in conidia. Collectively, these results corroborate that *FpvelC* was required for conidiation and spore wall remodeling in *F. proliferatum*.

To ensure the rapid infection and survival on hosts, the phytopathogens have evolved antioxidant enzymes to detoxify and quench reactive oxygen species (ROS) generated from plants, such as superoxide dismutase (SOD), catalase (CAT), peroxidase (POD) and ascorbate peroxidase (APX) [41]. NADPH oxidases are capable of univalent reduction of molecular oxygen into superoxide anion (O_2_^•−^), which is dismutated to H_2_O_2_ by SOD and subsequently converted into water by CAT [42]. It is worth noting that Δ*FpvelC* displayed significant sensitivity toward oxidative stress. Moreover, transcripts of NADPH oxidase subfamilies and catalase genes were decreased in Δ*FpvelC* after exposure to H_2_O_2_. These results might account for the possibility of *FpvelC* in modulating ROS scavenging. A similar phenomenon was reported in the maize pathogen *Fusarium verticillioides*, where velvet members VeA and VelB positively regulated the expression of catalase-encoding gene *FvCAT2* [43]. Once the encounter between hosts and pathogens occurred, plants produced ROS to eliminate pathogens or trigger immune responses [44]. How the pathogens utilize antioxidants to scavenge ROS from hosts might influence the outcomes of infections; the impaired capacity of the ROS scavenging system in Δ*FpvelC* cannot be ruled out, which was partially correlated with attenuated virulence against host challenges.

*F. proliferatum* employed a hemibiotrophic lifestyle to obtain host nutrients from living tissues before switching to a necrotrophic phase [27]. More notably, deletion of *FpvelC* in *F. proliferatum* displayed compromised colonization on rice and maize, which may be attributable to the pleiotropic factors. Firstly, the conidial production was dramatically decreased in Δ*FpvelC* compared with the Fp9 strain. Asexual spore was a primary inoculum that was extremely important for successful invasion of *F. proliferatum*. Upon landing on hosts, conidia germinated and subsequently colonized plant tissues. A similar observation was obtained from rice blast fungus *M. oryzae*, where *velC* positively regulated appressorial development; thus, loss of *velC* weakened the ability of appressorium to penetrate rice leaves [36]. By contrast, in the necrotrophic fungus *Botrytis cinerea*, disruption of *velC* did not affect infection on tomatoes and apples [45]. Secondly, the growth of invasive hyphae was found to be retarded in Δ*FpvelC*-infected rice glumes. After invasion into rice, *F. proliferatum* initially produced hyphal fragments to spread along the surfaces of plants [46]. Shortly thereafter, a switching of its lifestyle from biotrophy to necrotrophy occurred, thereby facilitating secondary hyphae to decompose or kill plant tissues [27]. As revealed, rice glumes inoculated with the Fp9 strain were filled with hyphae, causing the obvious breakage of cells, accompanied by abnormal accumulation of starch grains in chloroplasts. In contrast, *FpvelC* deficiency failed to form invasive hyphae; as a result, the development of starch grains was normal. Last but not least, expressions of genes encoding xylanases and xyloglucanases were downregulated in glumes infected with Δ*FpvelC*. Importantly, these enzymes were responsible for hydrolyzation of hemicellulosic components of plant cell walls, conferring an advantage for plant pathogens to hijack nutrient reservoir from the hosts [47], which were considered to be associated with pathogenicity in *B. cinerea* [48] and *V. dahliae* [49]. Therefore, we speculate that *FpvelC* regulated infection and host colonization by orchestrating conidia reproduction, development of invasive hyphae and secretion of extracellular enzymes in *F. proliferatum*.

The biosynthetic pathways of mycotoxins consisted of a cascade of intricate enzymatic reactions. These enzymes were encoded by genes in transcriptionally coregulated clusters, which tend to be located contiguously on the chromosome in *F. proliferatum* [27]. However, the elaborate mechanisms of how environmental and internal cues affected mycotoxin anabolism remain an enigma. Intriguingly, inactivation of *FpvelC* provoked production of FB1 and fusaric acid, which was in accordance with upregulation of biosynthetic clustering genes. To the best of our knowledge, this was the first report of negative regulation of *FpvelC* in fumonisins and fusaric acid in *F. proliferatum*. Nevertheless, in most fungal species, *velC* served as positive activators of the biosynthesis of secondary metabolites, such as kojic acid in *Aspergillus oryzae* [50], penicillin in *P. chrysogenum* [38] and patulin in *P. expansum* [23]. Peculiarly, lack of *velC* did not affect aflatoxins production in *A. flavus* or fumonisins biosynthesis in *F. verticillioides* [17,43]. Clearly, the discrepancies reveal that the roles of *velC* on secondary metabolism may be specific for each species. On the other hand, the phytotoxic activity of FB1 has been shown to contribute to virulence of *F. proliferatum* to rice [51]. However, loss of *FpvelC* enhanced FB1 production but did not accelerate exacerbation of disease development. Thus, we propose that FB1 was not essential for pathogenicity; it is plausible that additional determinants participated in the pathogenic diseases.

Nitrogen sources are indispensable elements for fungal growth and secondary metabolism. Nitrogen catabolite repression (NCR) allowed fungi to possess the capacity of preferential uptake of glutamine and ammonium [52]. In the rice *bakanae* disease pathogen *Fusarium fujikuroi*, the biosynthesis of fumonisins and fusaric acid was regulated by the quality and quantity of available nitrogen sources [52]. Unexpectedly, loss of function of *FpvelC* resulted in the upregulation of NCR regulators, ammonium permease and nitrate reductase, concomitant with a drastic reduction in the level of intracellular glutamine. This phenomenon can be interpreted by our previous study that FB1 production was specifically elicited by glutamine limitation, which was strictly dependent on NCR transcription factor FpareA in *F. proliferatum* [53]. The nucleus localization of FpareA responded to nitrogen starvation, leading to an elegant regulation of NCR-sensitive primary and secondary metabolism [53]. As a key metabolite in nitrogen metabolism, the signal of glutamine availability might be sensed by specific sensors. It is possible that loss of *FpvelC* gene resulted in glutamine limitation, which induced FpareA to be dephosphorylated and enter the nucleus, thereby driving expression of nitrogen catabolic genes. Moreover, GATA transcription factor AreA, AreA-binding protein Nmr and bZIP transcription factor MeaB were involved in the regulation of production of multiple secondary metabolites in *F. fujikuroi* [52]. Thus, we speculate that depletion of intracellular glutamine converged with *FpvelC* to coordinate FB1 biosynthesis. This idea is further supported by the fact that Δ*FpvelC* mutant exhibited apparent sensitivity after treatment with rapamycin (an inhibitor of TOR kinase). In eukaryotes, target of rapamycin (TOR) signaling responded to nutrients deficiency, and the TOR kinase activity became inactive under nitrogen starvation [54]. Thus, these findings provide evidence that *FpvelC* is involved in nitrogen sensing and signaling. A possible scenario is that *FpvelC* had inhibitory effects on biosynthesis of secondary metabolites, which might be connected with the crosstalk between nitrogen metabolism and TOR pathway.

## 4. Conclusions

In summary, this study highlighted that *FpvelC* positively governed conidiation, oxidative stress and virulence but negatively modulated production of fumonisins and fusaric acid. To the best of our knowledge, this was the first report where *FpvelC* was associated with mycotoxins biosynthesis in *F. proliferatum*. Our findings revealed the versatile roles of *FpvelC* in *F. proliferatum*, which contributed to the development of feasible strategies to mitigate the detrimental hazards of the pathogen on humans and plant hosts.

## 5. Materials and Methods

### 5.1. Fungal Strains and Growth Conditions

*F. proliferatum* strain Fp9 isolated from infected rice spikelets was used as the wild-type progenitor [27]. The Fp9 strain and its derivative transformants were stored as conidial suspensions at −80 °C with 30% (*v*/*v*) glycerol–water mixture. Fungal strains were cultured on potato dextrose agar (PDA, potato extract 200 g, dextrose 20 g and agar 15 g per liter) at 28 °C. For conidiation assays, the strains were incubated in yeast extract peptone dextrose (YEPD, yeast extract 10 g, peptone 20 g and dextrose 20 g per liter) on a rotary shaker for 3 days at 28 °C with 12 h light–dark cycle. The conidia were harvested by filtration and counted using a hemacytometer. For mycotoxin production, the strains were cultured at 28 °C in potato dextrose broth (PDB, potato extract 200 g and dextrose 20 g per liter). For determining the sensitivity of rapamycin, the strains were grown in minimal medium (MM, sucrose 30 g, glutamine 6 mM, KH_2_PO_4_ 1 g, MgSO_4_·7H_2_O 0.5 g, KCl 0.5 g, trace elements 200 μL and agar 20 g per liter) supplemented with rapamycin (Sigma-Aldrich, St. Louis, MO, USA) to the desired final concentrations. All experiments were performed at least three times.

### 5.2. Sequence Analysis of FpvelC

The amino acid sequence of FpvelC protein in *F. proliferatum* was identified by a BLASTP search at the National Center for Biotechnology Information (NCBI) using velC protein (ABQ17968) of *A. nidulans* as a query. The velvet domain was verified through the Conserved Domain Database at NCBI (https://www.ncbi.nlm.nih.gov/Structure/cdd/wrpsb.cgi) (accessed on 26 March 2024). The PEST motif was predicted using EMBOSS “epestfind” (http://emboss.bioinformatics.nl/cgi-bin/emboss/epestfind) (accessed on 26 March 2024). The available velC orthologs of different filamentous fungi were downloaded from the NCBI database. Multiple sequences were aligned with CLUSTALW [55]. A phylogenetic tree was constructed with the neighbor-joining algorithm with a bootstrap value of 1000 repetitions using the MEGA 11 software package (Mega Limited, Auckland, New Zealand) [56].

### 5.3. Gene Deletion and Complementation

Deletion and complementation of *FpvelC* gene were constructed by homologous recombination, following previously established protocols [57]. For gene deletion construction, the 5′- and 3′-flanking sequences were amplified with primers F1/R1 and F2/R2 from the genomic DNA of the Fp9 strain, respectively. Primers F3/R3 were used for amplification of hygromycin phosphotransferase gene (*HYG*) using plasmid pFGL821 as a template. Deletion fragments were generated with primers F4/R4 and F5/R5 by a double-joint PCR approach [58] and transformed into the protoplasts of Fp9 strain by polyethylene glycol (PEG)-mediated transformation. Positive transformants were selected on PDA plates containing 250 µg/mL hygromycin B (Calbiochem, La Jolla, CA, USA) and further identified by diagnostic PCR and Southern hybridization. For complementation construction, the full-length sequence of *FpvelC* gene containing the open-reading frame (ORF) region and its native promoter regions was amplified with primers Com-F1/Com-R1 from the genomic DNA of Fp9 strain. This fragment was fused with the geneticin resistance gene (*GEN*) and introduced into the protoplasts of Δ*FpvelC* mutants. The transformants were screened with 250 µg/mL G418 disulfate salt (Sigma-Aldrich, St. Louis, MO, USA) and verified by PCR analysis. The procedures of gene deletion and complementation are conducted as detailed in Figure S1 and Figure S2, respectively. All primers are listed with brief descriptions in Table S1.

### 5.4. Quantitative Real-Time PCR (qRT-PCR)

The total RNA was extracted using the TRIzol reagent (Invitrogen, Waltham, MA, USA) according to the manufacturer’s instructions. The quality and quantity of RNA were determined by a NanoDrop ND-1000 spectrophotometer (Nanodrop Technologies, Wilmington, DE, USA). First-strand complementary DNA (cDNA) was synthesized from total RNA with the QuantiTect Reverse Transcription Kit (Qiagen, Hilden, Germany). qRT-PCR reactions were performed with the SYBR Premix Ex Taq^TM^ II Kit (TaKaRa, Shiga, Japan) on a CFX96 Touch Real-Time PCR Detection system (Bio-Rad, Hercules, CA, USA). The *β*-tubulin (*Fptub*) gene of *F. proliferatum* was used as an internal control for normalization. The expression levels were calculated using the 2^−ΔΔCT^ method [59]. Transcript levels in Fp9 strain were arbitrarily set to 1. The specific primers used for gene expression analysis are listed in Table S2. The experiment was repeated three times with three technical replicates.

### 5.5. Trehalose Assay

The trehalose content was measured by the acid trehalase method [60]. Two-day conidia were collected by centrifugation, resuspended in sterile water at 1 × 10^7^ conidia/mL, and disrupted with Bullet Blender Gold homogenizer (Next Advance, Inc., Averill Park, NY, USA) at 4 °C for 10 min. The lysate was boiled at 95 °C for 20 min and centrifuged at 10,000× *g* for 10 min. The supernatant was digested with 3 mU of trehalose (Sigma-Aldrich, St. Louis, MO, USA) in 0.2 M sodium citrate buffer (pH 5.5) at 37 °C for 8 h. The reactions were stopped by the addition of 0.12 mol/L sulfuric acid. The amount of glucose generated from trehalose was examined with the Glucose Assay Kit (Sigma-Aldrich, St. Louis, MO, USA). Sample untreated with trehalase was a negative control. All experiments were conducted in triplicates.

### 5.6. Chitin Assay

The chitin content was estimated by assaying the release of glucosamine by hydrolysis of chitin [61]. Two-day conidia (1 × 10^8^) were harvested by centrifugation and incubated with lysis buffer (3% SDS, 0.3 M β-mercaptoethanol, 1 mM EDTA, 50 mM Tris, pH 8.0) at 100 °C for 40 min. The samples were washed three times with sterile saline solution and sonicated for 1 min on ice. After centrifugation at 10,000× *g* at 4 °C for 10 min, the pellet was acidified in 6 M HCl and hydrolyzed by N-acetylglucosamine (GlcNAc, Sigma-Aldrich, St. Louis, MO, USA) at 100 °C for 17 h. Samples were dried and dissolved in sterile water. Then, an equal volume of 4% (*v*/*v*) acetylacetone in 1.5 M Na_2_CO_3_ was added, the preparation was heated at 100 °C for 20 min. The samples were diluted with 96% ethanol and incubated with Ehrlich’s reagent buffer (26 mg/L p-dimethylaminobenzaldehyde, 5.8 M HCl, 50% ethanol) for 1 h at 20 °C. The absorbance at 520 nm was measured using a spectrophotometer (Genesys 10uv, Spectronic Unicam, Rochester, NY, USA). The experiment was repeated in triplicate.

### 5.7. Stress Tolerance Analysis

To assess responses to various stresses, the strains were grown on PDA media supplemented with chemicals, 0.3 mg/mL Congo red (Sigma-Aldrich, St. Louis, MO, USA) and 0.01% SDS for cell wall stress; or 1 mM or 5 mM H_2_O_2_ for oxidative stress. Strains without treatment were incubated in parallel as internal controls. After incubation at 28 °C for 5 days, the colonies were recorded and measured. The inhibition of mycelia growth was calculated using the formula [(C − N)/(C)] × 100%, where C is the colony diameter of the control and N is the colony diameter of the treatment. Each treatment consisted of three replicates, and the experiment was repeated thrice.

### 5.8. Rice Infection Assay

The susceptible rice cultivar Jiahe218 (China National Rice Research Institute, Hangzhou, Zhejing, China) was used for pathogenicity tests. Conidia were harvested from 3-day-old cultures in YEPD media and resuspended in sterile water to a final concentration of 1 × 10^6^ conidia/mL. At the booting stage, 1 mL of conidia suspension was injected into rice spikelets. The inoculated plants were maintained at 26 °C with a relative humidity of 85% and a photoperiod of 14 h in a greenhouse. Disease severity was assessed at 21 days post-inoculation (dpi) according to our previous method [62]. To further evaluate the infection behavior in florets, a 10 μL aliquot of conidial suspension was dropped into a floret at the anthesis stage. Infected rice glumes were observed under a scanning electron micrograph (SEM) and transmission electron micrograph (TEM), respectively. Ten plants were used for each strain, and the experiment was repeated three times independently.

### 5.9. Maize Infection Assay

The susceptible maize cultivar Suyunuo2 (Jiangsu Academy of Agricultural Sciences, Nanjing, Jiangsu, China) was selected for infection tests. The maize was surface-sterilized by 2% sodium hypochlorite (NaClO) and rinsed with sterile water three times. Maize kernels were aseptically wounded with sterile needles and inoculated with 10 μL of conidial suspension at a concentration of 5 × 10^6^ conidia/mL. Infected kernels were kept in a growth chamber at 28 °C with a relative humidity of 70%. Lesions were photographed at 7 dpi. Maize stems were punctured by sterile toothpicks to approximately 1 cm depth, and 50 μL of conidial suspension (5 × 10^6^ conidia/mL) was injected into the wounded stalks. Infected stalks were placed on wet filter papers in a sealed box. Stalk segments were dissected longitudinally, and the necroses were photographed at 14 dpi. All treatments were performed with at least three replicates, and each experiment was repeated three times.

### 5.10. Microscopy Observation

For scanning electron microscopy (SEM) examination, the samples were fixed in 2.5% glutaraldehyde solution overnight and washed three times with 0.1 M phosphate buffered saline (PBS). Samples were post-fixed in 1% osmic acid (Merck, Schwalbach, Germany) for 3 h and dehydrated in graded ethanol solutions (30%, 50%, 70%, 80%, 95% and 100% [*v*/*v*]) for 15 min with each grade. After critical point drying with liquid carbon dioxide, the specimens were sputter-coated with gold using a sputter coater (Bal-Tec SCD 005, Bal-Tec AG, Balzers, Liechtenstein) and visualized with a scanning electron microscope (Hitachi Model SU-8010, Hitachi High-Technologies Corporation, Tokyo, Japan) at an accelerating voltage of 2 kV. Three independent experiments were conducted with ten samples in each treatment.

For transmission electron microscopy (TEM) observation, the samples were fixed with 2.5% glutaraldehyde in 0.1 M sodium cacodylate buffer (pH 7.4) at room temperature for 2 h, fixed with 2% osmium tetroxide (Merck, Darmstadt, Germany) for 1 h at 4 °C. The samples were dehydrated in a series of ethanol (30%, 50%, 70%, 90% and 100% [*v*/*v*]) for 10 min each and embedded in Spurr’s resin (PolySciences, Niles, IL, USA). Ultrathin sections (80 nm) were sliced using an ultramicrotome (Leica EM UC6, Lecia Microsystems, Nussloch, Germany) and stained with 2% uranyl acetate and 0.4% Reynold’s lead citrate. The sections were examined with a transmission electron microscope (Hitachi Model H-7650, Hitachi High-Technologies Corporation, Tokyo, Japan) at an accelerating voltage of 120 kV. Each treatment contained ten samples, and the experiment was performed three times independently.

### 5.11. Determination of FB1 Production

Quantification of FB1 production was determined by HPLC-MS/MS [63]. The samples were collected from the fermentation broth in PDB and extracted with acetonitrile/water/acetic acid (74:25:1, *v*/*v*/*v*). The organic phase was filtered through a 0.22 μm polypropylene membrane. The chromatographic separation was performed with an HPLC system (HPLC-1260, Agilent Technologies, Santa Clara, CA, USA) on a Zorbax Extend-C18 column (100 × 2.1 mm, 3.5 μm). Mobile phases were composed of solvent A (water with 0.1% formic acid) and solvent B (methanol with 0.1% formic acid). The gradient elution was performed as follows: 0 to 1 min, 30% solvent B; 1.01 to 6.0 min, where solvent B was increased linearly from 30% to 80%; 6.01 to 9.0 min, where solvent B was maintained at 80%; 9.01 to 10.0 min, where solvent B was decreased linearly from 80% to 30%; and 10.01 to 16 min, where solvent B was maintained at 30%. A volume of 2 μL was injected into the column. The column oven was set at 30 °C. The flow rate was 0.3 mL/min. Mass spectra were analyzed in a triple quadrupole mass spectrometer equipped with an electrospray ionization (ESI) source in a positive ionization mode. Capillary voltages were kept at 3500 V. Nitrogen was supplied as the nebulizing and drying gas, the curtain gas was 18 psi, the nebulizer gas was 40 psi, the heater gas was 40 psi, and the CAD gas was 4 psi. The temperature of drying gas was set at 350 °C. The experiment was repeated three times.

### 5.12. Quantification of Fusaric Acid Production

Detection of fusaric acid was performed with the HPLC-MS/MS system [64]. The samples were harvested from PDB cultures and extracted with acetonitrile/water/acetic acid (79:20:1, *v*/*v*/*v*). The crude extract was centrifuged at 5000× *g* for 10 min, and the supernatant was filtered with a 0.45 μm polypropylene membrane. The chromatographic separation was carried out with a reversed-phase Zorbax SB-C18 column (150 × 4.6 mm, 5 μm, Agilent Technologies, Santa Clara, CA, USA). Mobile phases consisted of solvent A (0.1% trifluoroacetic acid in water) and solvent B (acetonitrile). The gradient elution procedure was as follows: 0 to 2 min, 10% solvent B; 2.01 to 20 min, where solvent B was increased linearly from 10% to 100%; and 20.01 to 28 min, where solvent B was maintained at 100%. The flow rate was 1 mL/min. The injection volume was 2 μL. The column oven was set at 45 °C. Mass spectra were operated in an ESI source in a positive ionization mode. The capillary voltage was kept at 3500 V, and the fragmentor voltage was set at 130 V. Nitrogen served as the curtain gas at 30 psi, and the nebulizer gas was set at 45 psi. The temperature of the drying gas was set at 350 °C. The experiment was conducted three times.

### 5.13. Measurement of Intracellular Glutamine

Glutamine content was analyzed by an ultra-high performance liquid chromatography system (UHPLC, 1290 Infinity, Agilent Technologies, Santa Clara, CA, USA) coupled with a tandem mass spectrometer (QTRAP5500, Applied Biosystems, Sciex, Concorde, ON, Canada) [65]. The samples were collected from mycelia and extracted with acetonitrile/water (1:1, *v*/*v*). After centrifugation, the supernatant was filtered with a 0.22 μm polypropylene membrane. Chromatographic separation was performed with a Waters ACQUITY UPLC BEH C18 column (2.1 × 150 mm, 1.7 μm, Waters, Milford, MA, USA). The column temperature was set at 35 °C. The flow rate was 0.3 mL/min and the injection volume was 2 μL. The composition of the mobile phases was solvent A (25 mM ammonium acetate and 0.08% formic acid in water) and solvent B (0.1% formic acid in acetonitrile). The linear gradient elution was as follows: 0 to 1 min, 85% solvent B; 1.01 to 11 min, where solvent B was decreased linearly from 85% to 50%; 11.01 to 12 min, where solvent B was maintained at 40%; 12.01 to 12.1 min, where solvent B was increased linearly from 40% to 75%; and 12.11 to 19 min, where solvent B was maintained at 75%. Mass spectrometry was equipped with an ESI source in positive ionization mode. The parameters were as follows: source temperature, 500 °C; ion source gas1, 55 psi; ion source gas2, 50 psi; curtain gas, 30 psi; ion spray voltage floating, 4500 V. The concentration of glutamine was expressed as μmol per g of dry weight of mycelia. The experiment was repeated thrice.

### 5.14. Statistical Analysis

All data are presented as the mean ± standard deviation of three individual replicates. Statistical analysis was performed using GraphPad Prism Version 7 (GraphPad Software Inc., San Diego, CA, USA). Significance was determined by a one-way analysis of variance (ANOVA) followed by Student’s *t*-test. Differences were considered to be statistically significant when the *p*-value was below 0.05.

## Data Availability

The original contributions presented in the study are included in the article/Supplementary Materials; further inquiries can be directed to the corresponding authors.

## Supplementary Materials

The following supporting information can be downloaded at: https://www.mdpi.com/article/10.3390/toxins17090433/s1, Figure S1: Schematic representation for the construction of the deletion mutant Δ*FpvelC*; Figure S2: Schematic representation of the construction of the complementation strain ∆*FpvelC*-C; Table S1: Primers used for gene deletion and complementation in this study; Table S2: Primers for qRT-PCR in this study.
