# Supplementary material for: Regulation of FpvelC on Conidiation, Pathogenicity and Secondary Metabolism in Fusarium proliferatum
Source: Toxins (Basel). 2025 Aug 30;17(9):433. doi: 10.3390/toxins17090433 (PMC12474422; doi:10.3390/toxins17090433)
Supplement: Supplementary file 1 [file toxins-17-00433-s001.zip › toxins-3818653-supplementary.pdf]

Supplementary Materials

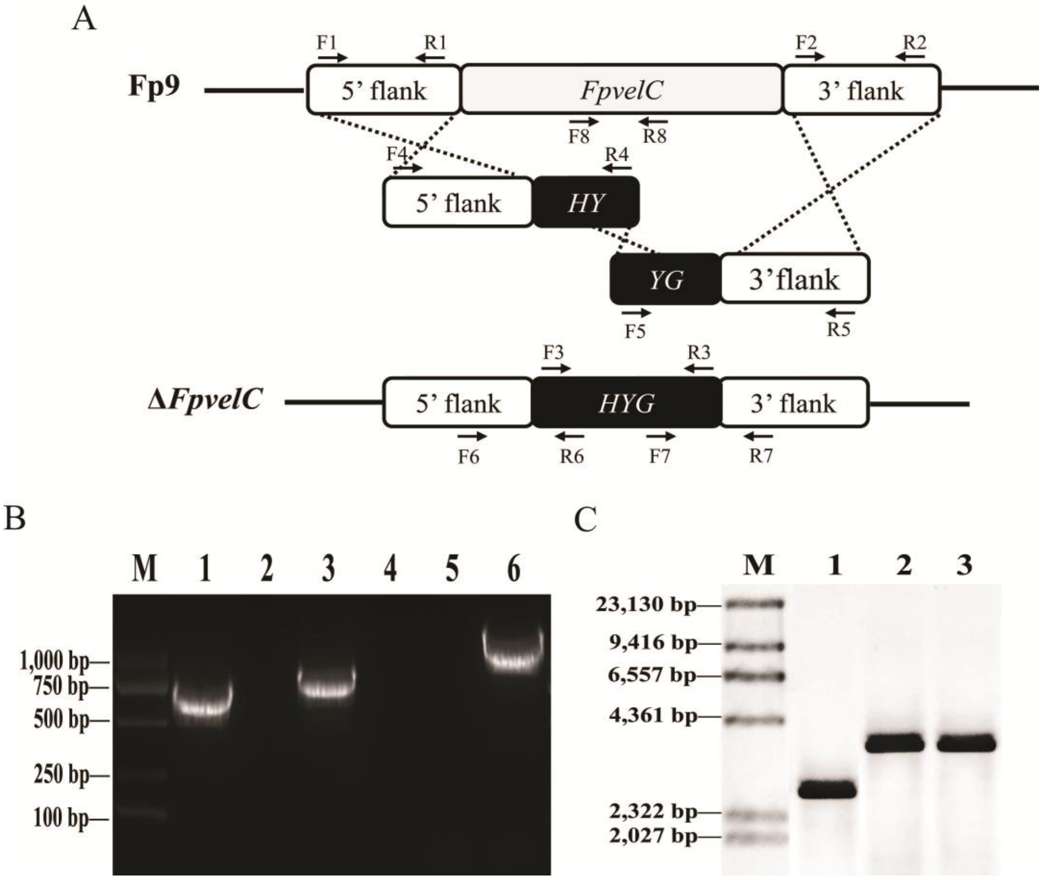

Figure S1. Schematic representation for the construction of deletion mutant Δ*FpvelC*. (A) Diagram of generation of deletion mutant Δ*FpvelC* via homologous recombination. *FpvelC* gene was replaced with hygromycin resistance cassette (*HYG*). *HY* showed 5'-flanking partial region of *HYG* gene, *YG* showed 3'-flanking partial region of *HYG* gene. The black arrows represented the position and orientation of the primers. (B) Diagnostic PCR analysis of deletion mutant Δ*FpvelC*. Primers F6/R6 and F7/R7 were used to amplify upstream and downstream regions of homologous recombination, while primers F8/R8 were used to amplify wild-type *FpvelC* allele. M: DL 2,000 DNA maker. Δ*FpvelC* mutant was detected in lanes 1, 3 and 5, and Fp9 strain was detected in lanes 2, 4 and 6. (C) Southern blot analysis of deletion mutant Δ*FpvelC*. Genomic DNA was digested with *Sac*I, separated on a 0.7% agarose gel, transferred to a nylon membrane and hybridized with DNA probe labeled by DIG. The upstream fragment of *FpvelC* gene was used as the probe. Expected bands were detected in Fp9 strain (lane 1) and Δ*FpvelC* mutants (lanes 2 and 3). Single-locus homologous recombination events occurred in Δ*FpvelC* mutants (lanes 2 and 3).

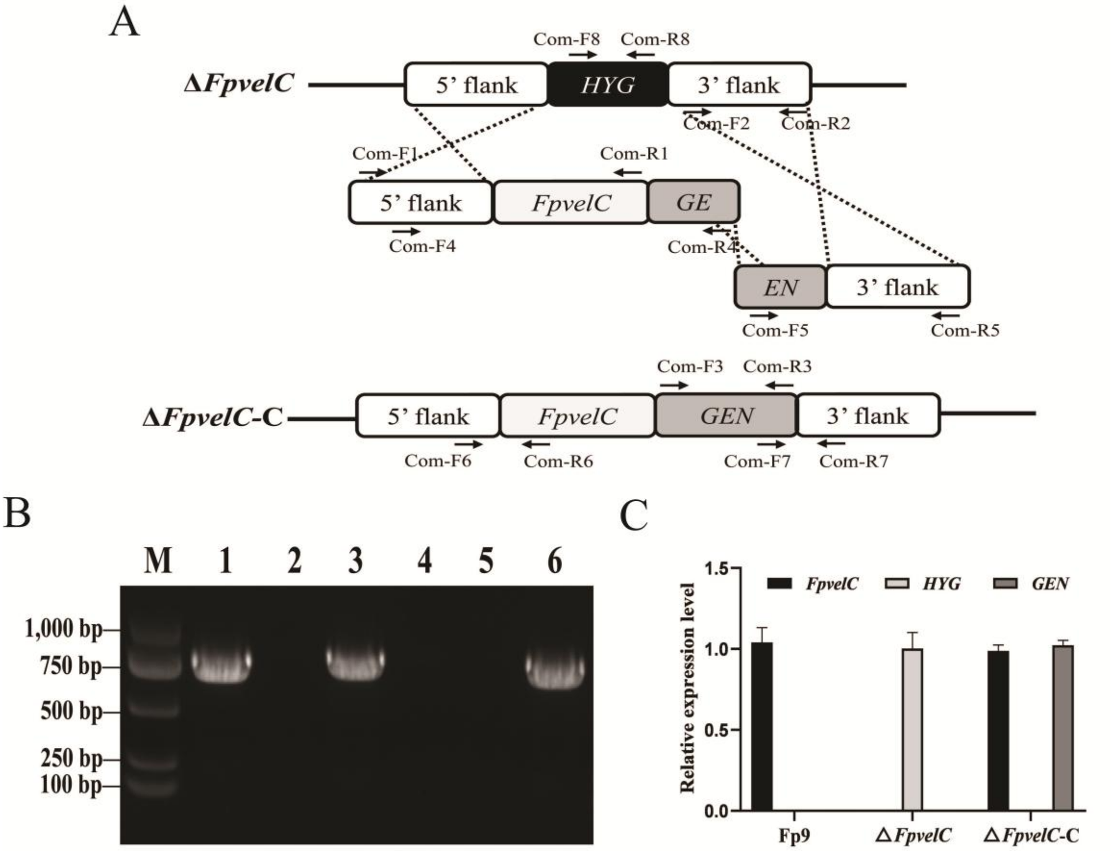

Figure S2. Schematic representation of the construction of complementation strain Δ*FpvelC*-C. (A) Diagram of generation of complementation strain Δ*FpvelC*-C. The *FpvelC* gene was transformed into the Δ*FpvelC* mutant to obtain Δ*FpvelC*-C. *GE* showed 5'-flanking partial region of *GEN* gene. *EN* showed 3'-flanking partial region of *GEN* gene. The position and orientation of the primers were indicated by horizontal arrows. (B) PCR amplification of the complementation strain Δ*FpvelC*-C. Primers Com-F6/Com-R6 and Com-F7/Com-R7 were used to amplify upstream and downstream regions of the complementation, while primers Com-F8/Com-R8 were used to amplify *HYG* gene. M: DL 2,000 DNA marker. Δ*FpvelC*-C was detected in lanes 1, 3 and 5, and Δ*FpvelC* was detected in lanes 2, 4 and 6. (C) Relative expression value of *FpvelC* gene by qRT-PCR. The expression of *β*-tubulin gene was used as an internal reference. Expression value of *FpvelC* gene in Fp9 strain was artificially set as 1. Bars represent standard deviation of three independent experiments with three technical replicates.

Table S1. Primers used for gene deletion and complementation in this study

Primers	Primer sequence (5′-3′)	Application	Purpose
F1	CGCAGCGAGGAAGTGACGCCTAAAATATATATTTTTTGATAGAAG	amplify *FpvelC* 5′ flank sequence	
R1	GCTTTTTCATACTCGCAGCATATAATAT	amplify *FpvelC* 5′ flank sequence	
F2	AAAGAAATAGTATTGTCGTCGCGTGTGG	amplify *FpvelC* 3′ flank sequence	
R2	CGCGCCGCCTGGCCTCGGTGGTACATACCAGAGATGAACCACTG	amplify *FpvelC* 3′ flank sequence	
F3	TGCTGCGAGTATGAAAAAGCCTGAACTCACCGC	amplify *HYG* sequence	Generation of deletion mutant
R3	GACGACAATACTATTTCTTTGCCCTCGGACG	amplify *HYG* sequence	
F4	AGCCCCAAGCCATCGCAA	amplify 5′ flank and *HYG* gene	
R4	CCAACCACGGCCTCCAGA	amplify 5′ flank and *HYG* gene	
F5	TATGTTTATCGGCACTTT	amplify 3′ flank and *HYG* gene	
R5	CCTTCGTTTTGACTTTTG	amplify 3′ flank and *HYG* gene	
F6	TGCCCCTTCTCAACATCA	Detection of left boarder of deletion mutants	
R6	ATCGCATCCATAGCCTCC	Detection of left boarder of deletion mutants	
F7	GCCGTGGTTGGCTTGTAT	Detection of right boarder of deletion mutants	
R7	CCCTTCCCGCTCTTTTCT	Detection of right boarder of deletion mutants	Confirmation of deletion mutant
F8	GGCATCAGAAGACCCAG	Detection of *FpvelC* gene of deletion mutants	
R8	CATCAACACCCACGAACT	Detection of *FpvelC*gene of deletion mutants	
F9	GCTTCGCCCTCATCTTG	Construction of DIG-labeled probe for Southern blot	
R9	TGCCTCAGAACCAACCC	Construction of DIG-labeled probe for Southern blot	
Com-F1	GGCAGAGCCGTGGGCCGACATCCCAAAACCTTGTGTCTCACT	amplify 5′ flank and *FpvelC* gene	
Com-R1	GTTCTTCTGAATGAATTGGGCACCGGACACA	amplify 5′ flank and *FpvelC* gene	
Com-F2	CAATCCCCATTATTGTCGTCGCGTGTGGA	amplify 3′ flank sequence	Generation of complementary strain
Com-R2	GCGGCCGGCCGGCGTGGTGGATATCCTTTAAGCTACCTGAGGT	amplify 3′ flank sequence	
Com-F3	CCCAATTCATTCAGAAGAACTCGTCAAGAAGGC	amplify *GEN* sequence	

Com-R3	GACGACAATAATGGGGATTGAACAAGATGGATTGC	amplify *GEN* sequence	
Com-F4	GGATGCCCACGGAAACCT	amplify 5′ flank, *FpvelC* and *GEN* gene	
Com-R4	TGCTCCTGCCGAGAAAGT	amplify 5′ flank, *FpvelC* and *GEN* gene	
Com-F5	CTCTTCAGCAATATCACG	amplify 3′ flank and *GEN* gene	
Com-R5	GCTCAACTCACTCACCTA	amplify 3′ flank and *GEN* gene	
Com-F6	GCCTTCATTCTGTATCGC	Detection of left boarder of complementation strains	
Com-R6	GGCTGCTTCTTCTGCTTT	Detection of left boarder of complementation strains	
Com-F7	GCATTTCGCTATTCCGTA	Detection of right boarder of complementation strains	Confirmation of complementation strain
Com-R7	CGCTTCCTCGTGCTTTAC	Detection of right boarder of complementation strains	
Com-F8	GTCCTGCGGGTAAATAGC	Detection of *HYG* gene of complementation strains	
Com-R8	AAATTGCCGTCAACCAA	Detection of *HYG* gene of complementation strains	

Table S2. Primers for qRT-PCR in this study

Gene name	Forward sequence (5'-3')	Reverse sequence (5'-3')	Annotation
Fpcon6	AGGCTACCATCAACAACCC	CGCCCATGACATCACCA	conidiation-specific protein
Fpcon7	CCTCAAGCACGACCAAA	ACATGCGCTCAATCTCC	conidiation-specific protein
Fpcon8	GCTCCTCAAACTGCTCCTAC	TCGCTTCTGTTCGCTGA	conidiation-specific protein
Fpcos1	TTCGCTGAGATTCCAAA	CAGCCCATACATGACCC	transcription factor
Fpcom1	GGGTGGTGTCACAAAGC	TCATGGTGCAGAGTCTCAT	transcription factor
Fptps2	TTGAGGACGACACGAAG	TGTGGGTAGTGCTGAACG	alpha,alpha-trehalose phosphate synthase
Fptps3	GAACTGGAAGCGAGGTG	GGAAAGCGACGTGTAAGA	alpha,alpha-trehalose phosphate synthase
FptreA	CTCACAACATCGCACCC	GAGAACCCGCGTAAGAA	alpha,alpha-trehalose glucohydrolase
FptreB	AAGGTCGTGGAGTGCCT	CTTCGCCGTGGTTGTAT	trehalase
FpchsE	GAGCCTTTCTGCGATTA	AGCAGTCAAGAGGTGGG	chitin synthase
FpchsZ	ACAAGAAATACGCAGAATG	CGCACAGTAAAGGACGA	chitin synthase
FpchiA	TCACCCTGACCCGCACTA	CCTCGTCAACGGCAGAAG	class III chitinase
FpchiB	CTTGATATTCGTGGAATGGA	GTTGTCGGGAGATGGGTA	class V chitinase
FpnoxA	GCATCCGTCAGCAGTCA	TCACGCACGAAACATCC	NADPH oxidase A
FpnoxB	AGGACTACATCTCGGTTCA	CGGGTTCCCTCTTCTTA	NADPH oxidase B
FpnoxR	TGTGAAGGAGAAGGTGGTC	GGCTTCGTTGGGTCTGT	NADPH oxidase R
FpcatA	AAGCCAGAGCCGCATAA	CGGTCAGACATAGCCCAC	catalase
FpcatD	GCTGGTGTTAAATCTTCTGT	GGATCGTCATCGTCGTA	catalase
Fpxyn10A	CCTCAGACCGAGCAGAC	GGATAGCATAGGTAGGGAAA	endo-1,4-beta-xylanase
Fpxyn10B	TTGGTATCACCGTCTGGG	AGGGCTTCTTCTGGAAGTTGT	endo-1,4-beta-xylanase
Fpxyn11B	CTACCTCGCTGTTTACGG	TGACCTCACCCTTCTTCT	endo-1,4-beta-xylanase
Fpxyn11C	TGGACCCGAAACCCTCT	TGCCATCAGCCTCAACG	endo-1,4-beta-xylanase
Fpxeg1	AACCGAAGGATACACCG	GGTAGACCACGAGAAGGAC	xyloglucanase
Fpxeg2	AGTCTATCCGCTTATCAACA	CTAACGCCGCTTTATTT	xyloglucanase

Fpxeg3	AGTTCCGGCTCTGGTTC	GTTGGGATACGCCTTGA	xyloglucanase
Fpxeg4	AGGTGATGACGGAGGTT	GATATTCCCATACTACAGACC	xyloglucanase
Fpfum1	CCAACTCTTCTTCCCTGCTA	CACCCTCTACCTCCCACA	polyketide synthase
Fpfum3	CCACGACCGATTTCACAG	TCCAGCCTTCATTTCAGC	dioxygenase
Fpfum6	CTGGAAAGTATGCGGTCAA	GCAGAACTCATCAGCGTCA	cytochrome P450 monooxygenase
Fpfum7	CAGGGCGGTTGACCACTT	GCGACGCCTGGATGTCTT	dehydrogenase
Fpfum8	GCGGAACGAGAAATAGTGA	TGCTGGGTTGAAAGGGAG	aminotransferase
Fpfum10	ACGGAATGACTGAGACGG	GGGAATCGGGTATTGACC	fatty acyl-CoA synthetase
Fpfum11	AAGGTGTTCTCGCCCTCT	ACCCTCCATCACTTTCTCAT	tricarboxylate transporter
Fpfum12	CGACGGCTACACTGCTTA	GCTCCTCACTAGACCCAAA	cytochrome P450 monooxygenase
Fpfum13	TCCGGGCAGCTCAGAATT	GGCGTGATACAGCGACCA	short-chain dehydrogenase/reductase
Fpfum14	TCAAGCTCGCCTCCTACCAC	CCACGATGACCGACTATCCC	peptide synthetase condensation domain
Fpfum15	AACCTCTACCCTATCTTCTGG	TGACTGTCTCCGTACCTGA	cytochrome P450 monooxygenase
Fpfum16	CCTTACAGATGCGTCCCT	ACCGCTTTCCTAATGGTC	fatty acyl-CoA synthetase
Fpfum17	TCTGGAGAAACCTCGAAAGG	ATGCCAATATGCGTGAAATG	longevity assurance factor
Fpfum18	TGATGTGAGGAGCGATGA	TCGAGATTCCTGCCAGCT	longevity assurance factor
Fpfum19	GGAGCCAGATTGGGACAG	ATACCCGAGGAGGAGCAG	ABC transporter
Fpfum21	CGACTGCCAGTATAAAGCC	GTAGCGTAACAGTTTGAGGAG	$Zn(II)_2Cys_6$ transcription factor
Fpfub1	TGATTGCCAATGGATCTCA	TATGCCTGGAGGGTTTGA	polyketide synthase
Fpfub2	AAGTCGCCGAGAATGGA	TCCGAAATCCCAAACCC	hypothetical protein of unknown function
Fpfub3	ACTTCGCAGTATCATTCG	TCTGCCGTGATTTGTCG	amino acid kinase
Fpfub4	CTCCCAAATCTCAAGCA	TCTGACTCGACGTAGGC	hydrolase
Fpfub5	CCTGTAGCCTTTGACCG	GTGAGGGCGATGTAGCA	acetyltransferase
Fpfub6	GCATTATCCCTAAAGAGC	CCAGCCAGACCAAGAGTA	dehydrogenase
Fpfub7	GTATCGGAGTCAAGGGAG	CAAGGGTGAATAGCAAGA	sulfhydrylase
Fpfub8	AAAAGTCCTGGAACGAA	GAAGCCATCAACAGTAGC	dehydrogenase

Fpfub9	GCGAGATCGCACCTATT	CAAAGACAAAGTCAGCACC	oxidase
Fpfub10	CATCCTCTGGTCCAAGTA	GCTGGCGTGTATGAAAA	C6 transcription factor
Fpfub11	AACCCAGTTCTCCCTCC	GATGCTCCTGCGTCTTT	major facilitator superfamily transporter
Fpfub12	GCAGCAACGCTTTAGAT	CCAAGAGGGCAGTGATAG	C6 transcription factor
FpareA	CAAATAGCACCAAACCAA	ACCCGATGTAGTACCACTTAC	GATA transcription factor
FpmeaB	GCAGCAACAGCAACAGA	CAGGAGTAGGGTAGTAAGAAG	bZIP transcription factor
Fpnmr1	CGCAAAGGAACTGACTGA	CAAGTAACGGGCTTCTCAT	NmrA family protein
Fpmep3	ACTCCTCCCAAGTCAGAAC	GCGGATTAGCACCTGTT	ammonium permease
Fpnii1	CGGCGAAGATACTGATG	CTGGCAACGTCCTTACA	nitrite reductase
Fptub	TCGGAAACTCCACCTC	GTCCATACCCTCACCAG	*β*-tubulin
